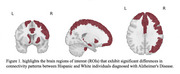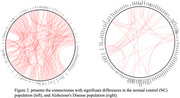# Brain Structural Network Alterations in Alzheimer's Disease: Perspectives from Ethnicity‐Specific Factors

**DOI:** 10.1002/alz70856_101821

**Published:** 2025-12-24

**Authors:** Haoteng Tang, Kun Zhao, Guodong Liu, Paul M. Thompson, Heng Huang, Alex Leow, Liang Zhan

**Affiliations:** ^1^ University of Texas Rio Grande Valley, Edinburg, TX, USA; ^2^ University of Pittsburgh, Pittsburgh, PA, USA; ^3^ University of Southern California, Los Angeles, CA, USA; ^4^ University of Maryland, College Park, MD, USA; ^5^ University of Illinois at Chicago, Chicago, IL, USA

## Abstract

**Background:**

Alzheimer's disease (AD) leads to distinct disruptions in brain networks, which can vary across ethnic populations. Using diffusion MRI‐derived brain structural networks, our study investigates ethnicity‐specific network alterations in Hispanic and White individuals during AD progression. Through network‐based statistical analysis, we identify significant subnetworks, including key brain regions‐of‐interest (ROIs) and connections, highlighting structural brain differences associated with ethnicity and disease status. These findings are critical for uncovering structural brain biomarkers and advancing our understanding of how demographic disparities influence neurodegenerative disease mechanisms, paving the way for more inclusive diagnostic and therapeutic strategies.

**Method:**

We utilize diffusion MRIs from OASIS (1326 subjects, mean age =70.42 ± 8.95, 738 women) and BrainLat dataset (763 subjects, mean age = 68.33 ± 11.19, 439 women) to construct structural brain networks (106 ROIs) based on Harvard‐Oxford Atlas and AAL Atlas. A Laplacian normalization is performed on these networks to standardize connectivity values, ensuring comparability across subjects and emphasizing relative structural differences by scaling node degrees and edge weights uniformly. Subjects are classified into AD and cognitively normal (NC) groups with specific ethnic labels (i.e., Hispanic and White). Network‐based statistic analysis was performed to identify significant subnetworks through edgewise comparisons between groups (e.g., AD/NC‐White vs. AD/NC‐Hispanic) with *p*‐value < 0.05/(106*105/2). This analysis focused on detecting ethnicity‐specific patterns in network topology and pinpointing significant ROIs/connectomes contributing to structural differences

**Result:**

A total of 167 significantly different connections involving 76 corresponding ROIs were identified between two ethnic groups in the NC population. For instance, the connections between the Frontal Pole and the posterior superior temporal gyrus, posterior middle temporal gyrus, anterior inferior temporal gyrus, and temporo‐occipital inferior temporal gyrus are stronger in the Hispanic group. In the AD population, 32 significantly different connections involving 35 corresponding ROIs were identified. For example, distinct connectomic patterns were observed among the Angular Gyrus, Temporal Fusiform Cortex, and hippocampal regions between AD‐White and AD‐Hispanic populations, which may be attributed to cultural influences and comorbidities such as vascular disease.

**Conclusion:**

We investigated ethnicity‐specific brain network differences using diffusion MRI in both NC and AD populations, identifying significant connectomic variations linked to different ethnicities.